# Overproduction of the cyanobacterial hydrogenase and selection of a mutant thriving on urea, as a possible step towards the future production of hydrogen coupled with water treatment

**DOI:** 10.1371/journal.pone.0198836

**Published:** 2018-06-07

**Authors:** Théo Veaudor, Marcia Ortega-Ramos, Thichakorn Jittawuttipoka, Hervé Bottin, Corinne Cassier-Chauvat, Franck Chauvat

**Affiliations:** Institute for Integrative Biology of the Cell (I2BC), CEA, CNRS, Univ Paris‐Sud, Université Paris‐Saclay, Gif‐sur‐Yvette, France; Albert-Ludwigs-Universitat Freiburg, GERMANY

## Abstract

Using a combination of various types of genetic manipulations (promoter replacement and gene cloning in replicating plasmid expression vector), we have overproduced the complex hydrogenase enzyme in the model cyanobacterium *Synechocystis* PCC6803. This new strain overproduces all twelve following proteins: HoxEFUYH (hydrogen production), HoxW (maturation of the HoxH subunit of hydrogenase) and HypABCDEF (assembly of the [NiFe] redox center of HoxHY hydrogenase). This strain when grown in the presence of a suitable quantities of nickel and iron used here exhibits a strong (25-fold) increase in hydrogenase activity, as compared to the WT strain growing in the standard medium. Hence, this strain can be very useful for future analyses of the cyanobacterial [NiFe] hydrogenase to determine its structure and, in turn, improve its tolerance to oxygen with the future goal of increasing hydrogen production. We also report the counterintuitive notion that lowering the activity of the *Synechocystis* urease can increase the photoproduction of biomass from urea-polluted waters, without decreasing hydrogenase activity. Such cyanobacterial factories with high hydrogenase activity and a healthy growth on urea constitute an important step towards the future development of an economical industrial processes coupling H_2_ production from solar energy and CO_2_, with wastewater treatment (urea depollution).

## Introduction

In response to the constant increase in energy consumption and resulting pollution by the growing and developping world population, it is important to develop new energy sources that are plentiful, renewable and environmentally friendly. Thus, the solar driven production of hydrogen (H_2_) is of special interest for many reasons. The annual solar flux received by Earth is in vast excess of the total energy used by human societies, and the burning of hydrogen liberates a high amount of energy (142 MJ/kg for H_2_ vs. 44.2 MJ/kg for oil) while producing only water (H_2_O) as a by-product. As a consequence, there is a growing interest in cyanobacteria, the photosynthetic microorganisms that can use solar energy, water, CO_2_, mineral and salts for the production of H_2_, while saving arable soils, fertilizers and fresh waters for agriculture (for recent reviews see [1–3]). Furthermore, several model cyanobacteria have a small sequenced genome easily manipulable, such as the presently studied unicellular strain *Synechocystis* PCC6803 (hereafter designated as *Synechocystis*). Its powerful genetics is necessary to attempt to increase the naturally low hydrogen production of cyanobacteria so as to reach levels that are of industrial interest.

The complex [NiFe] hydrogenase enzyme is well studied in *Synechocystis* where it is the sole enzyme capable of combining electrons with protons to produce H_2_ (*Synechocystis* has no nitrogenase enzyme) under specific conditions [1]. The pentameric hydrogenase enzyme (HoxEFUYH; Hox for hydrogen oxidation) has a bias toward H_2_ production [4], and is reversibly inactivated by oxygen [5].

The *Synechocystis* HoxEFUYH complex is encoded by the weakly expressed [6,7] *hoxEFUYH* operon encompassing the genes *hoxE*, *hoxF*, *sll1222*, *hoxU*, *hoxY*, *ssl2420*, *sll1225* and *hoxH* in that order (the function of *sll1222*, *ssl2420* and *sll1225* are not currently known). The HoxEFU diaphorase transfers the NAD(P)H-transported electrons originating from photosynthesis and/or sugar catabolism, to the [NiFe] hydrogenase sub-complex HoxHY. Interestingly, a recent *in vitro* analysis proposed that the hydrogenase enzyme could also be reduced by the electron carrier proteins ferredoxin or flavodoxin [8]. The [NiFe] HoxEFUYH complex is assembled by the six proteins encoded by the scattered *hypABCDEF* genes [1–3], while during HoxHY assembly, the C-terminus part of the HoxH subunit is cleaved off by the HoxW protease [9].

In an attempt to increase the low H_2_ production in *Synechocystis*, we previously characterized and then eliminated the AbrB2 repressor of the hoxEFUYH operon [6,10] with moderate success (two fold increase in hydrogenase activity). Other workers have replaced the weak promoter of the *hoxEFUYH* operon by the light-inducible promoter of the photosynthetic gene *psbAII* and obtained a three-fold increase in H_2_ production [11]. Similarly, we replaced the promoter of the *hoxEFUYH* operon by the strong (lambda-phage) promoter λ*p*_R_. We also cloned the *hypABCDEF* genes under the control of the λ*p*_R_ promoter of our overexpression vector derived from the RSF1010 promiscuous plasmid capable of replication to the same copy number (10 per cell) as the chromosome [12,13]. The resulting mutants strongly overproduced HoxEFUYH proteins both alone or in combination with HypABCDEF but showed limited increase in hydrogenase activity [14]. These findings showed that increasing the abundance of the HoxEFUYH and HypABCDEF proteins is not sufficient to strongly increase hydrogenase activity. One factor possibly limiting the hydrogenase activity of our mutants overproducing HoxEFUYH without or with HypABCDEF, could be the natural level of the HoxW protein needed to mature HoxH (via cleavage of its *C*-terminus) [9,15,16]). Thus, it is possible that the natural level of HoxW could be insufficient to mature the increased abundance of HoxH. To test this, we constructed and analyzed strains that simultaneously overproduce HoxEFUYH and HoxW, alone or with HypABCDEF. We report here that the largest increase is conferred by the simultaneous overproduction of HoxEFUYH and HoxW together with HypABCDEF. This hydrogenase overproducing strain is of great interest for future biochemical and structural studies of the cyanobacterial [NiFe] hydrogenase. To the best of our knowledge, a high-resolution structure of the cyanobacterial [NiFe] hydrogenase remain to be determined. In turn, this information would be of great help to design a meaningful strategy to engineer an oxygen-resistant hydrogenase for the over- photoproduction of H_2_.

In addition to the level of production *per se*, one potential approach to limit the cost of H_2_ production is to couple it with the treatment of wastewaters, which are often polluted with urea originating from mammalian wastes or its use as an agricultural fertilizer [17]. Most organisms that can use urea (CO(NH_2_)_2_) as a nitrogen source employ a nickel-dependent metalloenzyme called urease (EC 3.5.1.5, also named urea amidohydrolase) to catalyze the ATP-independent hydrolysis of urea into ammonia (NH_3_) and carbon dioxide (CO_2_) [18]. Furthermore, the urease activity consumes no reducing power, unlike the nitrate reductase enzyme. Hence, these spare electrons can be used for H_2_ production driven by the HoxEFUYH enzyme. In most bacteria and cyanobacteria, urease is a trimer of three subunits (UreA, UreB and UreC) that requires up to four (accessory) chaperone proteins (UreD, UreE, UreF and UreG) for activation and incorporation of two nickel atoms into its metallocenter active site [18]. In this study, we tested the influence of urea on the production of biomass and hydrogenase activity in wild-type and hydrogenase overproducing strains. We show here that (i) decreasing the activity of urease is beneficial for cell growth on urea as the sole nitrogen source, and (ii) the activity of hydrogenase was similar in cell growing on urea or nitrate. These novel results and strains have important implication for the future production of H_2_ from solar energy, CO_2_ and urea-polluted waters.

## Materials and methods

### Bacterial strains and growth conditions

*Synechocystis* PCC6803 (*Synechocystis*) was grown at 30°C under continuous white light (2,500 lux; 31.25 μE m^-2^ s^-1^) on the mineral medium BG11 [19] enriched with 3.78 mM Na_2_CO_3_ [20] hereafter designated as MM. For some experiments MM was supplemented with 1–2.5 μM NiSO_4_ and/or 17 μM Fe (provided as green ferric ammonium citrate) and/or nitrate was replaced by urea (5 mM) and/or ammonium chloride (1–20 mM) as indicated. In these latter cases nitrate-grown cells were washed twice with sterile water before resuspension in urea- and/or ammonium-containing media).

*E*. *coli* strains used for gene manipulations (DH5α or TOP10, Invitrogen®) or conjugative transfer [12,13] to *Synechocystis* (CM404) of replicative plasmids derived from our expression vector pFC1 (S1 Table) were grown on LB medium at 37°C (TOP10 and DH5α), or at 30°C (CM404 and TOP10 cells harboring pFC1 derivatives). Antibiotic selections were as follows for *E*. *coli*: ampicillin (Ap) 100 μg.ml^-1^, gentamycin (Gm) 10 μg.mL^-1^, kanamycin (Km) 50 μg.ml^-1^ and spectinomycin (Sp) 100 μg.ml^-1^; for *Synechocystis*: Gm 2.5–5 μg. mL^-1^, Km 50–300 μg.ml^-1^, Sp 2.5–5 μg.ml^-1^ and Sm 2.5–5 μg.ml^-1^.

### Gene cloning and manipulation

*Synechocystis* DNA was PCR amplified with specific primers (S2 Table) to generate the studied genes that were either overexpressed or eliminated. For gene overexpression experiments, the protein coding sequences were cloned downstream of the strong λ*p*_R_ promoter and associated ribosome binding site of our replicative plasmid vector derived from the broad-host-range plasmid RSF1010 [13]. The resulting plasmids were introduced by conjugation in *Synechocystis* [12]. For gene deletion, the genomic regions encompassing each studied gene were PCR amplified, or synthezised (Eurofins), prior to replacement by an antibiotic resistance marker for selection. The resulting deletion cassettes (S1 Table) were introduced by transformation into *Synechocystis* [21] where homologous DNA recombination between the deletion cassette and the recipient chromosome integrated the antibiotic resistant marker in place of the studied gene.

All DNA constructions were verified through PCR and DNA sequencing (Big Dye kit, ABI Perkin Elmer, or Eurofins), before and after propagation in *Synechocystis*. PCR was also used to test whether the segregation between the mutant (antibiotic resistant) and WT copies of the polyploïd chromosome (about 10 per cell, [21] was complete (the studied gene is dispensable to cell growth) or not (the gene is essential to cell viability).

### RNA isolation and analysis by quantitative RT-PCR

Exponentially growing cells were rapidly harvested and broken (Eaton press), prior to RNA purification and analysis by quantitative RT-PCR using primers (S2 Table) that generated DNA fragments of similar size (163–234 bp), as previously described [6,14]. Each assay was performed in triplicate to allow the calculation of the mean threshold cycle value (*C*_*T*_) for each studied gene, which was converted to gene copy number per ng of template cDNA.

### Western blot analysis of the HoxF and HoxH proteins

40 μg of *Synechocystis* proteins were separated, transferred to nitrocellulose membrane (Invitrogen) and immunodetected with rabbit anti-HoxF and anti-HoxH antibodies, and the R800 goat anti-rabbit IgG secondary antibodies, as previously described [14].

### Hydrogenase activities

They were measured by the standard amperometric method in an anaerobic glove box equipped with an inverted Clark-type electrode (Hansatech, UK), using Na-dithionite (20 mM, reducing agent) and methylviologen (5 mM, electron donor) as previously described [6,14].

### Urease activities

They were monitored by the release of ammonia from urea [22]. Cells collected by centrifugation (5,000 rpm, 10 min, 20°C) were resuspended in 400–1000 μL of buffer (PBS 1X pH 7.5, EDTA 10 mM, Protease Inhibitor Cocktail, Roche) and disrupted in a pre-cooled Eaton press, as described [14]. 10 μg of total proteins (measured with the Bradford protein assay, Biorad) were mixed with 90 μL of PBS EDTA buffer lacking (reaction blank) or containing (samples) 50 mM urea and then incubated for 10 min at 30°C. Tubes were transferred on ice before the addition of 150 μL of phenol nitroprusside (Sigma-Aldrich), 150 μL of alkaline hypochlorite (Sigma-Aldrich) and 700 μL H_2_O. Chromophore formation was achieved at 37°C for 10 min before measuring absorbance at 570 nm. After substracting the A_570_ values of blank reactions, the ammonia concentration of the samples was determined from a standard curve constructed with varying NH_4_Cl concentrations.

### *In silico* modeling and docking simulations

Each amino acids (AA) sequence was modelled with the intensive search mode of Phyre2 [23]. Poorly modelled AA residues or regions were manually removed with Swiss PDB Viewer. For each structure, a Ramachandran plot was generated using Chimera to verify that less than 5% of residues were modelled with conflicting sidechain orientations. Reliability of models was further evaluated using ModFOLD, ModEval and Qmean servers [24–26]. Multimeric complexes were obtained with Chimera by superposition of finihed models to published oligomeric structures.

## Results

### Construction and analysis of a mutant for high level expression of all hoxEFUYHW genes from the strong lambda-phage p_*R*_ promoter

**Fig 1:**
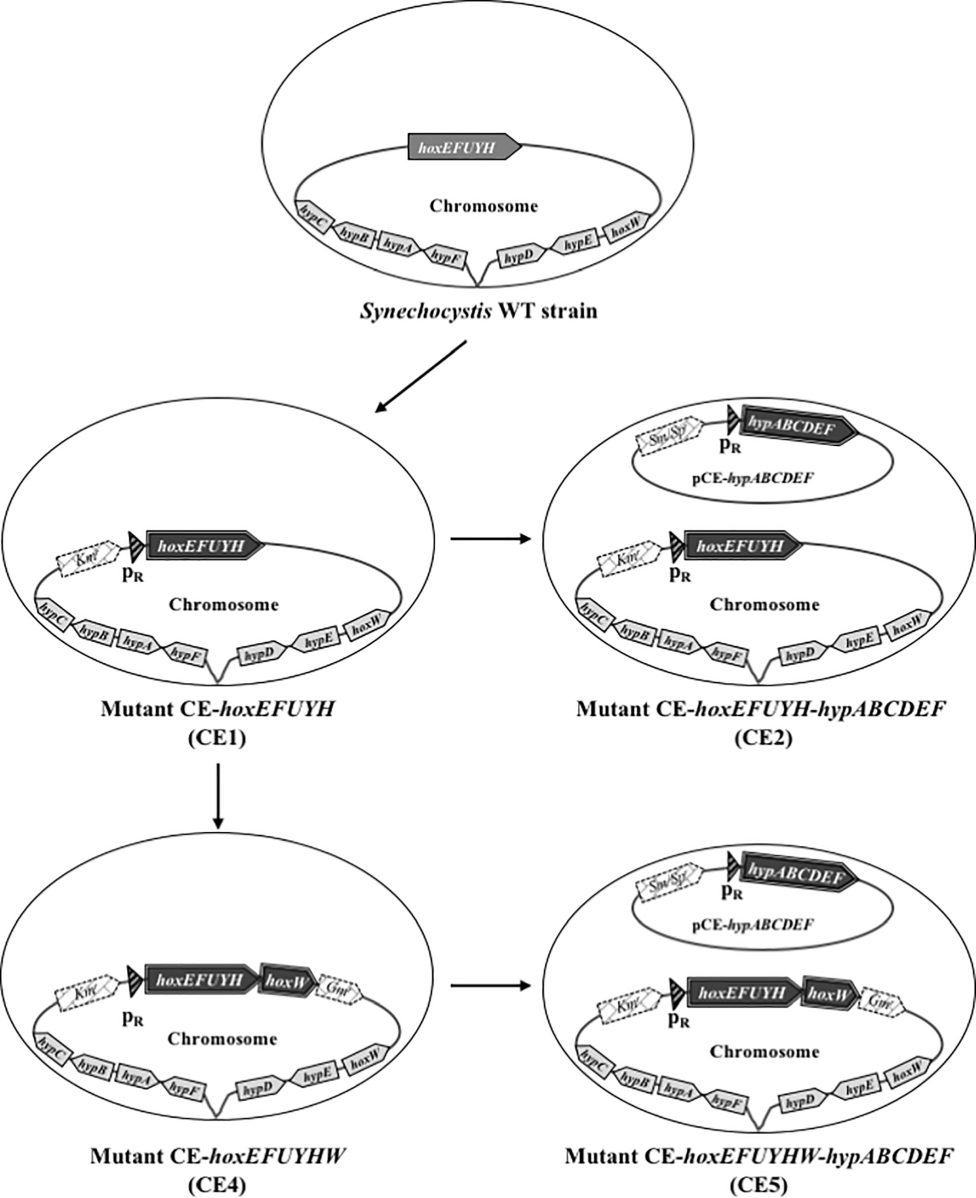
Schematic representation of the *Synechocystis* strains constructed in this study. *Synechocystis* cells are represented by oval shapes showing their chromosome attached to the cell membrane. The pCE-*hypABCDEF* replicating plasmid is represented by circles. The *hoxEFUYHW* and *hypABCDEF* genes and the antibiotic resistance markers are shown by large arrows pointing towards the direction of their transcription. The triangle represents the strong lambda phage pR promoter (λ*p*_R_); CE for strong constitutive expression.

**Fig 2:**
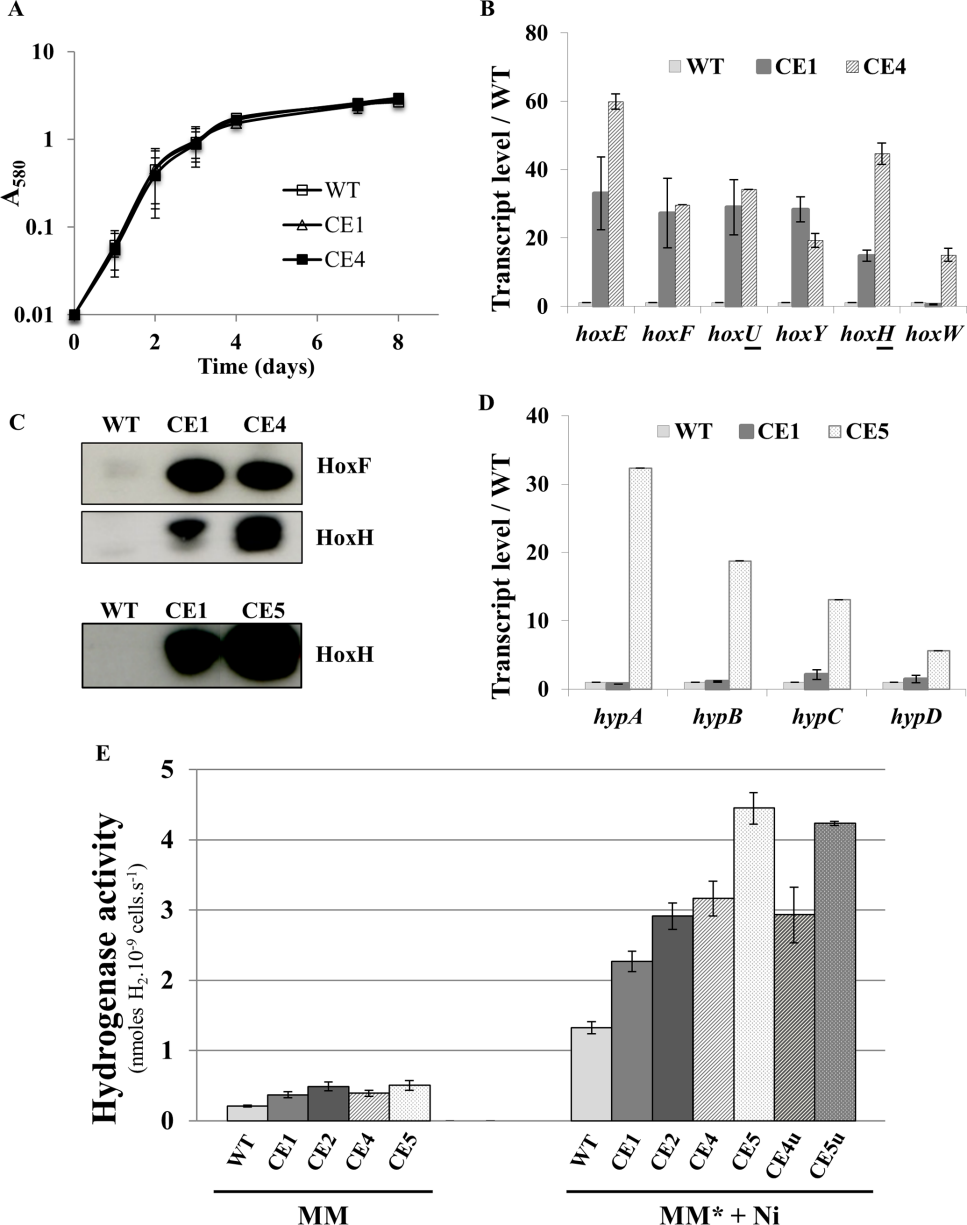
Comparative analysis of the strains over-expressing the genes *hoxEFUYH* (CE1) or *hoxEFUYHW* (CE4; CE4u) alone, or together with the *hypABCDEF* genes (CE5; CE5u). All experiments were performed at least three times. (**A**) Typical growth of the wild type (WT; squares), CE-*hoxEFUYH* (CE1; white triangles) and CE-*hoxEFUYHW* (CE4; black squares) cells incubated under standard conditions. (**B**) Histogram plot representation of transcript abundance (measured by Real-time quantitative PCR) of the *hoxEFUYHW* genes in strains WT (small light-grey bars), CE1 (grey rectangles) and CE4 (hatched bars) mutants. (**C**) Western blot analysis of the abundance of the HoxF and HoxH proteins in WT, CE1, CE4 and CE5 cells growing in MM* medium (MM + 17 μM Fe). (**D**) Histogram plot representation of the transcript abundance (RT-qPCR) of the *hypABC-F* genes in the strains WT (light-grey rectangles), CE1 (grey) and CE5 (arrow-filled bars). (**E**) Histograms representation of the hydrogenase activities of WT (light grey), CE1 (grey), CE2 (dark grey) CE4 (light grey-hatched bars), CE5 (white arrow-filled bars), CE4u (dark grey-hatched bars) and CE5u cells (grey arrow-filled bars) growing in standard medium (MM) or MM* (MM + 17 μM Fe) supplemented with 2.5 μM NiSO_4_.

In our previous attempt to engineer *Synechocystis* for high-level photoproduction of H_2_, we increased the expression of the *hoxEFUYH* operon by replacing its weak natural promoter [6] by the strong λ*p*_R_ (lambda-phage) promoter [27], thereby yielding the CE-*hoxEFUYH* mutant also called CE1 (CE for constitutive strong expression) [14] and this work (Fig 1). The CE1 mutant over-producing the *hoxEFUYH* transcripts (at least 100-fold) and HoxEFUYH proteins exhibited only a small increase in hydrogenase activity as compared to wild-type (WT) cells [14] and this work (Fig 2). We also found that the hydrogenase activity of both WT and CE1 cells could be increased by growing the cells in the mineral medium appropriately supplemented with extra iron (17 μM) and nickel (2.5 μM) metals required for the [NiFe] hydrogenase cluster. Furthermore, the gains conferred by the genetic engineering and growth-medium improvement could be cumulative as the hydrogenase activity of the CE1 mutant grow in the improved medium was about 10-fold higher than hydrogenase activity of WT cells grow in the standard medium cells [14] and this work (Fig 2). However, this increase in hydrogenase activity was modest in comparison to the large increase in abundance of the HoxEFUYH transcripts and proteins in the CE1 mutant (see [14] and this work (Fig 2)). Thus, we hypothesized that the natural level of the HoxW protease needed for the proteolytic maturation of the natural quantity of HoxH, might be limiting in CE1 cells that overproduce the HoxEFUYH proteins. This assumption is consistent with the finding that several cyanobacteria (*Synechococcus* PCC7335, *Halothece* PCC741 and *Leptolyngbya* PCC7375) harbor several copies (2–3) of *hoxW*, while they maintain *hoxEFUYH* as single copy genes [3]. To further increase the level of hydrogenase activity of our CE1 mutant we cloned the *hoxW* protein-coding-sequence, downstream of the chromosomal *hoxEFUYH* operon expressed from the strong [27] λ*p*_R_ promoter. To achieve this, a *hoxW*-Gm^r^ DNA cassette was constructed by PCR in such a way that it was flanked by appropriate *Synechocystis* DNA sequences to serve as regions of homology for DNA recombinations promoting the introduction of the *hoxW*-Gm^r^ cassette downstream of *hoxH* (S1 Fig). The resulting *hoxW*-Gm^r^ cassette was verified by PCR (S2 Fig) and nucleotide sequencing, and subsequently transformed [21] to CE1 cells (Km^r^), yielding Km^r^-Gm^r^ clones. This Km^r^-Gm^r^ mutant grew as well as the WT strain in standard photoautotrophic conditions (Fig 2). This mutant was analyzed by PCR (S2 Fig) in order to verify that the *hoxW* protein-coding-sequence was successfully integrated downstream of the *hoxH* gene of the *hoxEFUYH* operon, generating the *hoxEFUYHW* operon, as expected. Furthermore, we found that all copies of the *Synechocystis* polyploid chromosome [21], harbor the *hoxEFUYHW* operon. This mutant was designated as CE4 (constitutive strong expression of the *hoxEFUYHW* genes, Fig 1). We also verified by PCR that CE4 cells possessed only CE4-type (*hoxEFUYHW*-Gm^r^) chromosome copies, even after growth in the absence of Gm to allow the possible re-establishment of any remaining CE1 (Gm^s^) chromosome copies (S2 Fig). We then verified, using quantitative RT-PCR, that the CE4 strain produced similar high levels of *hoxEFUYH* transcripts as the CE1 mutant, and more abundant (about 20-fold) *hoxW* mRNA than WT and CE1 cells (Fig 2). Collectively, these data demonstrate that the overexpression of the *hoxEFUYHW* genes is not detrimental to cell viability, in agreement with the absence of negative physiological effects of the overexpression of the *hoxEFUYH* genes, on one hand (CE1 cells [14] and (Fig 2)), and *hoxW* on the other hand (Fig 2). Indeed, we also cloned *hoxW* just behind the strong [27] λ*p*_R_ promoter of the plasmid vector for maximal expression (pFC1ΔcI_857_, S1 Table), and found that the resulting cells grew very well (data not shown).

As expected, the hydrogenase activity of CE4 cells was higher than those of CE1 and WT cells, in that order (Fig 2), irrespectively of the use of the standard MM medium or the improved medium supplemented with Fe (17 μM) and Ni (2.5 μM) metals (the Hox hydrogenase uses a [NiFe] redox center). The higher hydrogenase activity of CE4 (overexpression of *hoxE****F****UY****H****W*), as compared to CE1 (overexpression of *hoxE****F****UY****H***), was consistent with the positive role of HoxW on hydrogenase activity (proteolytic maturation of the HoxH subunit [9]). This finding indicates that the increased expression of *hoxW* in CE4 was necessary to yield a large quantity of mature HoxH, and by extension the natural (WT) level of HoxW in CE1 cells was insufficient to cleave the large amount of HoxH to maturity. This interpretation was also supported by the western blot analysis of the HoxF and HoxH proteins (Fig 2). In WT cells, the very faint HoxF and HoxH signals were consistent with the low abundance of these proteins in *Synechocystis* seen with Western blots and quantitative proteomics [14]. Furthermore, in CE1 cells (Fig 2), the more abundant bands on Western blots of HoxF and HoxH (mostly the large HoxH form not yet cleaved by the low natural quantity of HoxW) also confirmed our previous report [14]. Collectively, the present data (Fig 2) show that CE4 cells (overexpression of *hoxE****F****UY****H****W*) produce similar high quantities of HoxF and HoxH as CE1 (overexpression of *hoxE****F****UY****H***). The thicker HoxH signal observed in CE4, as compared to CE1, is likely due to the increased abundance of the small form of HoxH cleaved by the increased level of HoxW.

### Construction and analysis of a mutant for strong constitutive expression of all *hoxEFUYHW* and *hypABCDEF* genes in *Synechocystis*

In a previous study, we showed that the gain in hydrogenase activity conferred by the overproduction of the hoxEFUYH proteins can be further increased by the simultaneous overproduction of the HypABCDEF proteins directed by the (Sm^r^/Sp^r^) plasmid pCE-*hypABCDEF* [14]. This pCE-*hypABCDEF* vector, derived from the promiscuous plasmid RSF1010 that replicates autonomously to the same 10–20 copies per cell as the chromosome [13], strongly express the *hypABCDEF* gene from the very-active λ*p*_R_ promoter [14]. Consequently, we decided to simultaneously overproduce all HoxEFUYHW proteins and all HypABCDEF proteins. To accomplish this, we introduced the Sm^r^/Sp^r^ pCE-*hypABCDEF* plasmid by conjugation [12] into the Km^r^-Gm^r^ CE-*hoxEFUYHW* (CE4) mutant (Fig 1). This yielded the CE-*hoxEFUYHW*-*hypABCDEF* mutant (Fig 1) designated as CE5 (note that CE5 cells also carry the weakly expressed WT alleles of *hoxW* and *hypABCDEF* in their chromosome). CE5 cells grew as well as WT cells (data not shown) and CE4 cells in standard photoautotrophic conditions (Fig 2). As expected, the CE5 strain strongly expressed the *hoxEFUYHW* and *hypABCDEF* genes (Fig 2) and its hydrogenase activity was higher than that of the CE4, CE1 and WT strains, in that order (Fig 2). Altogether, the constitutive overexpression of the *hoxEFUYHW* and *hypABCDEF* genes, and the increased Ni- and Fe-availabilities led to the strongest increase in active hydrogenase (25-fold) as compared to WT cells cultivated in normal medium (Fig 2).

### Influence of urea and urease activity on cell growth and hydrogenase activity

**Fig 3:**
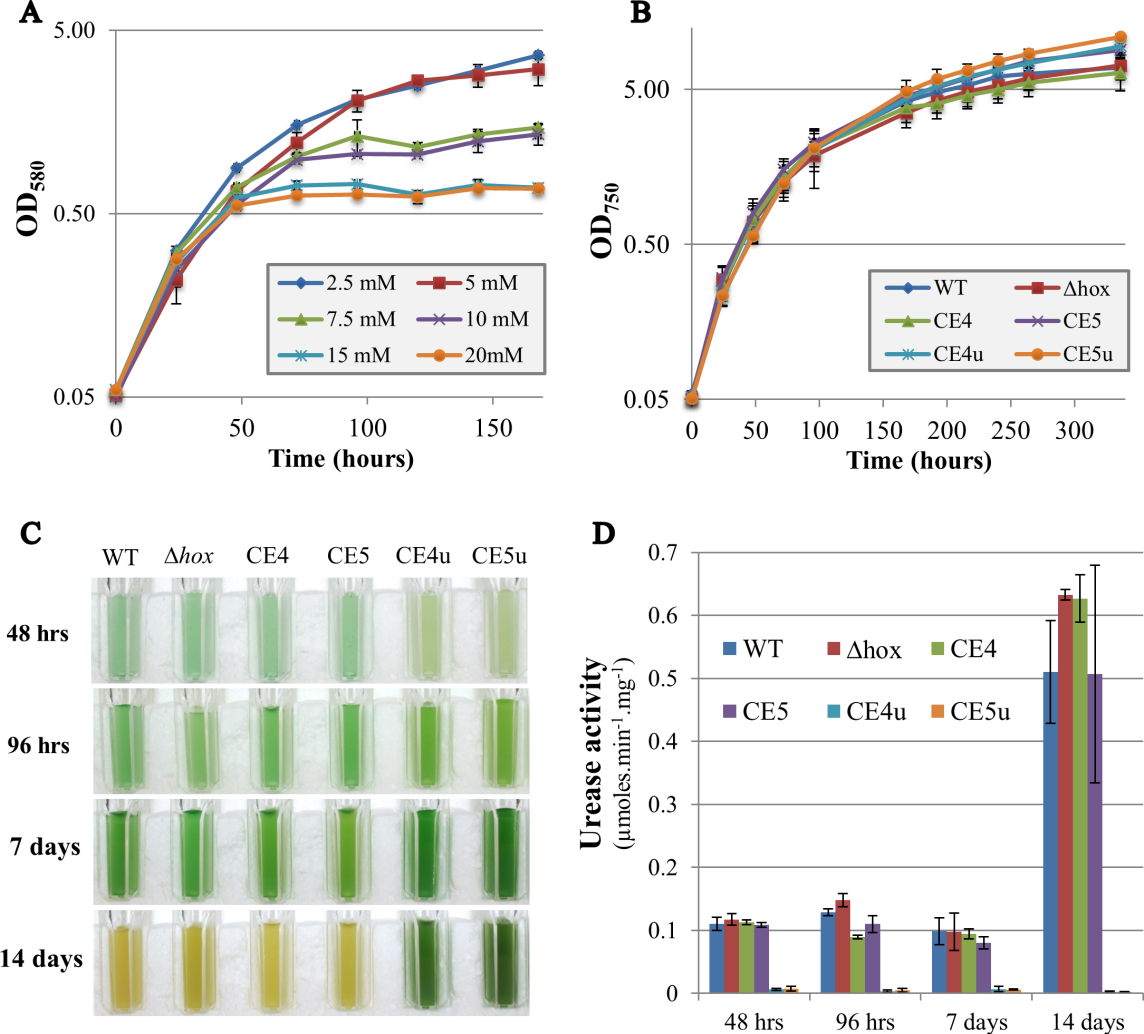
Influence of urea on the growth of *Synechocystis* WT and mutants overexpressing the *hoxEFUYHW* genes alone (CE4) or in combination with the *hypABCDEF* genes (CE5). (**A**) Typical growth of WT cells cultivated on medium containing Ni (1 μM) and urea (2.5–20 mM) as the sole nitrogen source. (**B**) Typical growth on urea (5 mM as the sole nitrogen source) and Ni (2.5 μM) of the WT strain, and the CE4 and CE5 strain without or with (CE4u and CE5u) a mutation in *ureG*. Influence of prolonged growth on urea (5 mM as the sole nitrogen source) and Ni (2.5 μM) on the cell appearance **C**) and urease activity (**D**) of the studied strains. All experiments were performed at least three times.

As a step towards the future development of an economic cyanobacterial process for the photoproduction of H_2_ coupled with wastewater treatment, we have tested the influence of urea (a frequent water pollutant) on the biomass production and hydrogenase activity of our strains. Therefore, *Synechocystis* WT was incubated for increasing periods of time in liquid mineral medium containing urea as the sole nitrogen source, and nickel (both urease and hydrogenase require Ni). WT cells grew well on urea up to 5 mM, whereas higher urea concentrations reduced the duration of healthy growth and production of biomass (Fig 3). We then tested the influence of 5 mM urea on the growth and hydrogenase activity of the WT and CE1-CE5 strains, and the previously constructed delta *hoxEFUYH* mutant [14]. All these strains grew well and displayed the normal blue green color for more than 7 days (Fig 3). During this time, the hydrogenase activity of the WT and CE5 strains did not vary (data not shown).

After 14 days of cultivation on urea (5 mM, sole N source) and Ni (2.5 μM) cells reaching the stationary phase of growth (OD_750_ > 5.0) became invariably yellowish (Fig 3). Once the chlorosis process became visibly detectable, a large decrease in cell viability was observed by plating assay on solid standard growth medium (it contains nitrate, not urea).

A similar urea-induced chlorosis was observed in the cyanobacteria *Anabaena cylindrica* and *Synechococcus* PCC7002, distantly related to *Synechocystis* PCC6803, when they were cultivated on urea as the sole nitrogen source [28,29]. The urea-mediated chlorosis and cell death were found not to occur in a urease defective mutant harboring an inactivated allele of *ureC*, the gene normally encoding the large urease subunit. To test whether the same is true in *Synechocystis*, we inactivated its *ureC* gene by replacing its first 971 bp by the Km^r^ marker gene (S3 Fig), yielding the λ*ureC*::Km^r^ DNA cassette. Following transformation to *Synechocystis* [21], Km^r^ clones growing in standard medium (i.e. on nitrate as the sole nitrogen source) supplemented with Km were analyzed by PCR to verify that all chromosome copies harbor the Δ*ureC*::Km^r^ cassette in place of *ureC* (S3 Fig). The complete absence of WT (*ureC*^+^) chromosome copies in the Δ*ureC*::Km^r^ mutant was confirmed by analyzing cells that were subsequently grown in absence of Km to allow a possible re-invasion of *ureC*^+^ chromosome copies if any remained. We also verified that the Δ*ureC*::Km^r^ mutant had lost the urease activity and the capability to grow on urea as the sole nitrogen source (data not shown). These results demonstrate that the *Synechocystis ureC* gene is essential for urease activity and cell growth on urea as the sole nitrogen source, as observed in the distantly related cyanobacteria *Synechococcus* PCC7002 [29] and *Synechococcus* PCCWH7805 [30].

During our study, we found one mutant clone of each hydrogenase overproducing strains CE4 and CE5 that grew stably on urea as the sole N source and retained the normal blue-green color. These clones escaping the urea-promoted chlorosis (Fig 3) were named CE4u and CE5u (u for withstanding urea as the sole nitrogen source). Interestingly, the CE4u and CE5u strains retained their high hydrogenase activity (Fig 2). Knowing that the urea-mediated chlorosis depends on an active urease (see above), we confirmed that the urease in the CE4u and CE5u strains were less effective than the enzyme in the WT, CE4 and CE5 strains (Fig 2). To identify the mutation in CE4u and CE5u responsible for their low urease activity, we used specific oligonucleotide primers for PCR amplification and nucleotide sequencing of all seven urease genes (*ureABCDEFG*) including their upstream and downstream regions (about 200 bp in each case). We also used qRT-PCR to monitor and compare the expression of the *ureABCDEFG* genes in CE4u and CE5u (urea-tolerant) and WT (urea-sensitive) strains. The results showed that the expression of *ureABCDEFG* was not affected in CE4u and CE5u (S4 Fig and data not shown). Interestingly, both CE4u and CE5u carried the same (single) mutation, a C to T transition at position 254 of the *ureG* coding sequence, which substituted the alanine amino-acid residue at position 85 by a valine residue (mutation A85V; S4 Fig). To confirm that the low urease activity of CE4u is due to the A85V mutation in *ureG*, we performed a plasmid complementation test. We cloned the WT *ureG* protein-coding-sequence in the replicative plasmid vector for strong constitutive gene expression (S5 Fig). The resulting plasmid pCE-*ureG* was introduced by conjugation in the CE4u strain, which harbors the *ureG*_A85V_ gene in its chromosome, and in the WT, CE1 and CE4 control strains, which have *ureG* in their chromosome. As expected, in the CE4u/pCE-*ureG* test strain, the production of the wild-type UreG protein directed by the pCE-*ureG* plasmid increased the urease activity (3-fold) well above the level observed in the CE4u strain producing only the UreG_A85V_ mutant protein (S4 Fig). In contrast, the control strains WT/pCE-*ureG*, CE1/pCE-*ureG* and CE4/pCE-*ureG*, which produced UreG from both their chromosome and their pCE-*ureG* plasmid, exhibited no increase in urease activity when compared to their plasmid-free parental strains WT, CE1 and CE4, which produced UreG only from their chromosome. It appeared that the stronger level of *ureG* expression driven by both the plasmid and the chromosome slightly decreased the overall urease activity for an unknown reason (S4 Fig; compare WT/pCE-*ureG* with WT).

The alanine 85 of UreG belongs to an α-helix situated near the Ni-binding site of UreG, which physically interacts with the groove formed by the UreF dimer (S4 Fig). It is thus possible that the steric hindrance generated by the A85V mutation in ureG somehow impaired the UreG-UreF physical interaction, thereby decreasing the incorporation of Ni atoms in the urease active site. Collectively, these findings showed that UreG plays a crucial role on urease activity of *Synechocystis* as observed in the evolutionary distant cyanobacterium *Anabaena* PCC7120 [Valladares, 2002 #108]. Interestingly, a similar alanine to valine mutation (A142V) near the Ni-binding of the soybean UreG (A142 corresponds to G64 in the *Synechocystis* protein) was also shown to decrease urease activity [31].

Finally, we found that hydrogenase activity is not affected by the decline in urease activity (Fig 2) that allows cell to grow stably on media containing urea as the sole nitrogen source.

## Discussion

We showed here that the simultaneous overproduction of all HoxEFUYHW and HypABCDEF proteins involved in the synthesis, maturation and assembly of the [NiFe] hydrogenase complex, combined with media improvement, led to a strong (about 25-fold) increase in the level of active hydrogenase. We also showed the counterintuitive notion that lowering the activity of the *Synechocystis* urease can improve the photoproduction of biomass, and in turn H_2_, from urea-polluted waters. The sophisticated strains constructed during this work displayed a higher increase in expression of the *hoxEFUYH* and *hypABCDEF* genes than that of hydrogenase activity. This finding indicates that limiting post-transcriptional factors need to be dealt with in order to engineer a powerful H_2_ producer of industrial importance. We think that the presently described strain with a healthy growth and an increased abundance of active hydrogenase is a suitable starting point for this important objective. Indeed, this hydrogenase overproducing strain should facilitate the purification and structural analysis of hydrogenase (it structure is as yet unknown), which, in turn, should facilitate the design of meaningfull strategies to increase its (low) tolerance to O_2_ that is massively produced by photosynthesis. Furthermore, recent *in vitro* data suggested that the hydrogenase enzyme could receive electrons not only from NAD(P)H but also the well conserved electron transfer proteins [32] ferredoxins [8]. Thus, it will be interesting to try to further increase the hydrogenase activity of our high-hydrogenase level strain by overproducing each of the nine *Synechocystis* ferredoxins [32] along with its HoxEFUYHW and HypABCDEF proteins. It will also be important to examine the redox state of the cysteine amino-acids of the HoxH and HoxF subunits, since it has been shown that they can be oxidized [33,34], a finding that should stimulate the analysis of crosstalks between hydrogen production and redox (oxidative) stress [1].

Towards a future objective of reducing the cost of H_2_ photoproduction by combining it with the treatment of water, we chose to test the influence of urea, a frequent pollutant [17], on *Synechocystis* growth (production of biomass) and hydrogenase activity. We found that *Synechocystis* coud grow for several days on urea (5 mM) as the sole nitrogen source, but invariably cells turned yellow and died upon longer incubation times (for example in 14 days, Fig 3). Furthermore, cells were killed faster by higher urea concentration (7.5–20 mM; the higher the concentration the faster was cell death; Fig 3). Similar findings were observed in the cyanobacteria *Anabaena cylindrica* and *Synechococcus* PCC7002 [28,29] phylogenetically-distant from *Synechocystis*. Urea-stressed *Synechococcus* PCC7002 cells were shown to contain high levels of lipid peroxides. Furthermore, exogenously added polyunsaturated fatty acids triggered a similar death response, while vitamin E suppressed the formation of peroxides and delayed the onset of chlorosis and cell death. These results suggest that cyanobacterial cells grown on urea for several days undergo a metabolic imbalance that ultimately leads to oxidative stress and lipid peroxidation. As observed in *Synechococcus* PCC7002 [29] we found that that the inactivation (deletion in our study) of the *Synechocystis ureC* gene, normally encoding a catalytic subunits of urease, impaired urease activity, as well as cell growth on urea and subsequent urea-induced chlorosis and cell death (Fig 3). We also found that lowering the activity of the urease enzyme (for example with the UreG_A85V_ mutation) can sustain *Synechocystis* growth on media containing urea as the sole nitrogen source. Furthermore, we found that the urea tolerance mutation had no detrimental effect on hydrogenase activity. The urea-tolerant hydrogenase-overproduction strain displayed the same hydrogenase activity level in both media containing either nitrate or urea as the sole nitrogen sources. Altogether the present findings imply that it should be possible to reduce the cost of hydrogen production by combining it with water treatment (urea depollution) in the future.

## Conclusion

The presently reported cyanobacterial factories with a large hydrogenase activity and healthy growth on urea will be very useful for the purification of large hydrogenase quantities for biochemical and structural analyses, in order to better understand this important enzyme and improve its (weak) tolerance to O_2_. Such work has interesting implications for future economically viable industrial production of H_2_ from solar energy, CO_2_ and urea-polluted waters.

## Supporting information

S1 Fig Construction of the *hoxW*-Gm^r^ DNA cassette for cloning *hoxW* behind *hoxH*, the last gene of the hoxEFUYH operon. The genes are represented by large arrows pointing towards the direction of their transcription. The black square indicates the ribosome binding site introduced in front of the *hoxW* coding sequence. The grey rectangle shown as TT designates the transcription and translation stop signals (TT), which prevent read-through of gene expression from the Gm^r^ marker. The hoxH-up and HoxH-dwn DNA regions served as platform for homologous recombinations that occurred during transformation and led to the introduction of the *hoxW*-Gm^r^ DNA cassette behind the *hoxEFUYH* operon.
(TIFF)

S2 Fig PCR verification of the CE-*hoxEFUYHW* mutant (CE4) for strong constitutive expression of the *hoxEFUYHW* genes. (**A**) Schematic representation of the *hoxEFUYH* operon in the WT strain and the CE4 mutant (CE-*hoxEFUYHW*) which contains the Km^r^-λ*p*_*R*_ DNA cassette in place of the weak promoter of the *hoxEFUYH* operon, and the *hoxW*-Gm^r^ DNA cassette behind the *hoxEFUYH* operon. The oligonucleotide primers represented by small colored triangles (S2 Table) served for the PCR verifications indicated by double arrows. (**B**) UV-light image of the agarose gel showing the 0.54 kb and 1.9 kb PCR DNA products typical of the WT strain and the CE4 mutant, respectively. Marker (M) = GeneRuler™ 1Kb Plus DNA Ladder (Fermentas). The lane noted H_2_O correspond to a negative control with no DNA-template.
(TIFF)

S3 Fig Inactivation of the *ureC* gene. (**A**) Construction of the Δ*ureC*::Km^r^ DNA cassette. The genes are represented by large arrows pointing towards the direction of their transcription. The ureC-up and ureC-dwn DNA regions served as platform for homologous recombinations promoting the targeted replacement of *ureC* by the Km^r^ gene, upon transformation to *Synechocystis*. (**B**) Schematic representation of the *ureC* locus in the wild-type strain (WT) and the Δ*ureC*::Km^r^ mutant, which harbors the Km^r^ marker in place of the first 971 bp of the *ureC* coding sequence. The blue and red triangles represent the oligonucleotides primers that generated the PCR DNA segments (double arrows) typical of the WT strain or the Δ*ureC*::Km^r^ mutant. (**C**) UV-light image of the agarose gel showing the PCR products typical of the chromosome organization in the WT strain and the Δ*ureC*::Km^r^ mutant growing in standard conditions. Marker (M) = GeneRuler™ 1Kb plus DNA Ladder (Fermentas). The lane noted H_2_O served as a negative control (no DNA template) while pEX-A *ureC*::Km^r^ served as a positive control for the three Δ*ureC*::Km^r^ mutants clones cultivated in the presence (MM + Km) or absence (MM) of kanamycin.
(TIFF)

S4 Fig Influence of the spontaneous single mutation in *ureG* coding sequence (A85V) of the CE4 clone (termed CE4u) on the expression of the urease genes and urease activity. (**A**) Positions of the nucleotide (blue) and amino-acid (red) sequence of the relevant part of *ureG* showing the mutation (red rectangle) and conserved nickel binding site (orange rectangle). (**B**) Histogram representation of transcript abundance (RT-qPCR) of the urease genes in the WT strain (blue rectangles) and CE4u (hatched-green bars) mutant. (**C**) Urease activity of the WT, CE1, CE4 and CE4u strains (blue, red, green and hatched-green rectangles, respectively) lacking (noted as -) or containing (+) the pCE-*ureG* replicative plasmid that overexpresses ureG. (**D**) Structural models of the *Synechocystis* urease accessory proteins (chaperones) UreG and UreF. Subunits of the UreG dimer (top) are represented in their GDP-bound form and colored in light brown and grey, respectively. The mutated A85 residues are highlighted by thick green coils while GDP molecules are depicted in blue. Nickel-binding residues (Cys and His) are represented as sticks. Black arrows point to the G64 residues (Ni binding site). Front (bottom left) and side (bottom right) views of the UreG dimer interacting with the UreF subunits, colored in orange and red, respectively.
(TIFF)

S5 Fig Construction of the pCE-*ureG* plasmid for strong expression of the *ureG* gene. (**A**) Schematic representation of the *Synechocystis* chromosome region harboring *ureG*. The genes are represented by large arrows pointing towards the direction of their transcription. The pFC1 expression vector [13]was digested by *Psi*I cand religated to inactivate the temperature-dependent λ*c*I_857_ repressor gene, which normally controls the strong λ*p*_R_ promoter (red triangle), yielding pFC1Δ*c*I_857_ (also called pCE, CE for constitutive expression). Meanwhile, ureG was amplified with oligonucleotides primers that introduced a *Nde*I site (embedding its ATG start codon) and an *Eco*RI site (behind its stop codon). After *Nde*I/*Eco*RI double digestion ureG was cloned between the *Nde*I and *Eco*RI sites of pCE, yielding the pCE-*ureG* plasmid. (**B**) UV-light image of the agarose gel showing the PCR products typical of the pCE-*ureG* plasmid replicating in the WT, CE1, CE4 and CE4u strains. Marker (M) = GeneRuler™ 1Kb plus DNA Ladder (Fermentas). The lane noted H_2_O served as a negative control (no DNA template) while those noted pFC1Δ*c*I_857_ and pCE-*ureG* served as a positive control of the presence of the corresponding plasmid in the studied *Synechocystis* strains (two clones analyzed in every case).
(TIFF)

S1 Table Characteristics of the plasmids used in this study. CS, Protein Coding Sequence; Δ, deletion; TT, transcriptional terminator.
(DOCX)

S2 Table List of the PCR primers used in this study. The restriction sites are written in bold letters; CS, coding sequence; RBS, ribosome binding site. Fw and Rv in the primers names stand for “forward” and “reverse”, respectively.
(DOCX)
