# Supplementary figures and images for: Overproduction of the cyanobacterial hydrogenase and selection of a mutant thriving on urea, as a possible step towards the future production of hydrogen coupled with water treatment
Source: PLoS One. 2018 Jun 7;13(6):e0198836. doi: 10.1371/journal.pone.0198836 (PMC5991728; doi:10.1371/journal.pone.0198836)

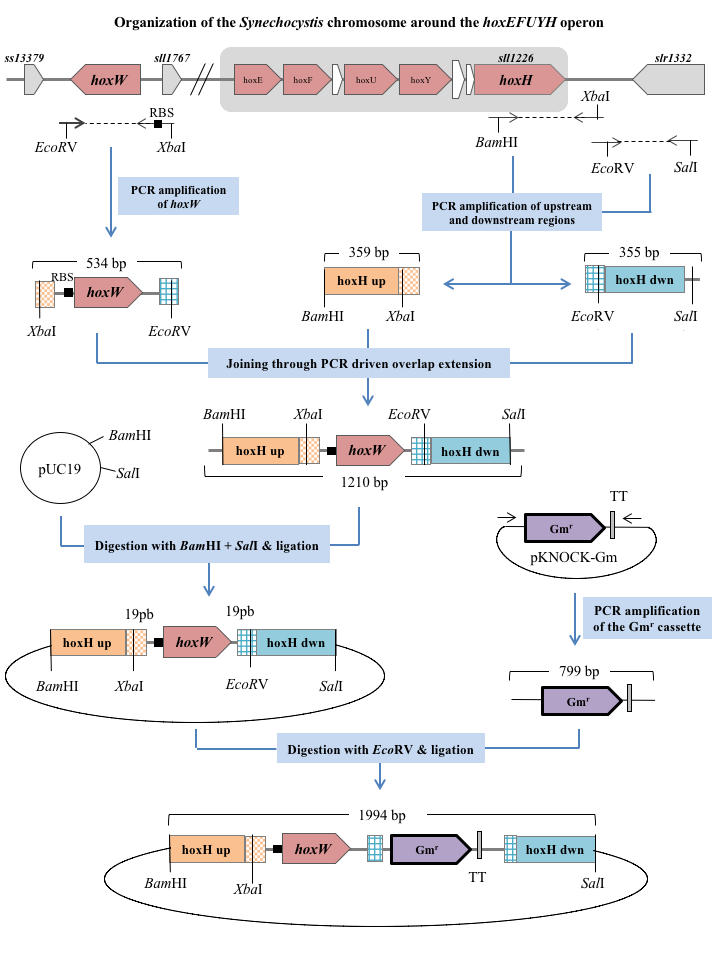

Supplement: S1 Fig — The genes are represented by large arrows pointing towards the direction of their transcription. The black square indicates the ribosome binding site introduced in front of the hoxW coding sequence. The grey rectangle shown as TT designates the transcription and translation stop signals (TT), which prevent read-through of gene expression from the Gmr marker. The hoxH-up and HoxH-dwn DNA regions served as platform for homologous recombinations that occurred during transformation and led to the introduction of the hoxW-Gmr DNA cassette behind the hoxEFUYH operon. (TIFF) [file pone.0198836.s001.tiff]

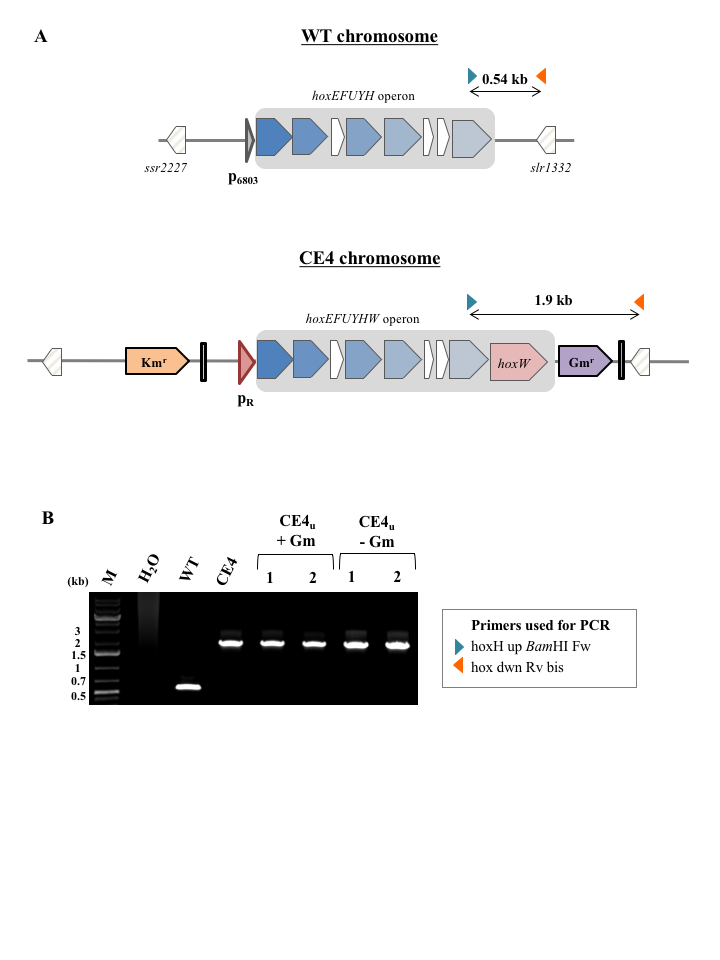

Supplement: S2 Fig — (A) Schematic representation of the hoxEFUYH operon in the WT strain and the CE4 mutant (CE-hoxEFUYHW) which contains the Kmr-λpR DNA cassette in place of the weak promoter of the hoxEFUYH operon, and the hoxW-Gmr DNA cassette behind the hoxEFUYH operon. The oligonucleotide primers represented by small colored triangles (S2 Table) served for the PCR verifications indicated by double arrows. (B) UV-light image of the agarose gel showing the 0.54 kb and 1.9 kb PCR DNA products typical of the WT strain and the CE4 mutant, respectively. Marker (M) = GeneRuler™ 1Kb Plus DNA Ladder (Fermentas). The lane noted H2O correspond to a negative control with no DNA-template. (TIFF) [file pone.0198836.s002.tiff]

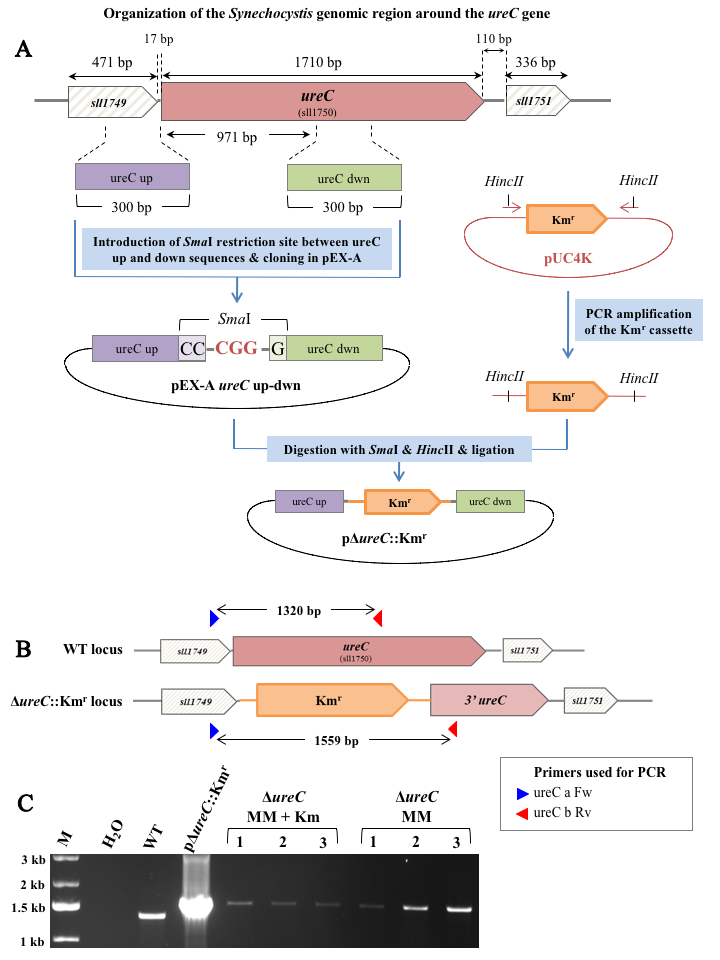

Supplement: S3 Fig — (A) Construction of the ΔureC::Kmr DNA cassette. The genes are represented by large arrows pointing towards the direction of their transcription. The ureC-up and ureC-dwn DNA regions served as platform for homologous recombinations promoting the targeted replacement of ureC by the Kmr gene, upon transformation to Synechocystis. (B) Schematic representation of the ureC locus in the wild-type strain (WT) and the ΔureC::Kmr mutant, which harbors the Kmr marker in place of the first 971 bp of the ureC coding sequence. The blue and red triangles represent the oligonucleotides primers that generated the PCR DNA segments (double arrows) typical of the WT strain or the ΔureC::Kmr mutant. (C) UV-light image of the agarose gel showing the PCR products typical of the chromosome organization in the WT strain and the ΔureC::Kmr mutant growing in standard conditions. Marker (M) = GeneRuler™ 1Kb plus DNA Ladder (Fermentas). The lane noted H2O served as a negative control (no DNA template) while pEX-A ureC::Kmr served as a positive control for the three ΔureC::Kmr mutants clones cultivated in the presence (MM + Km) or absence (MM) of kanamycin. (TIFF) [file pone.0198836.s003.tiff]

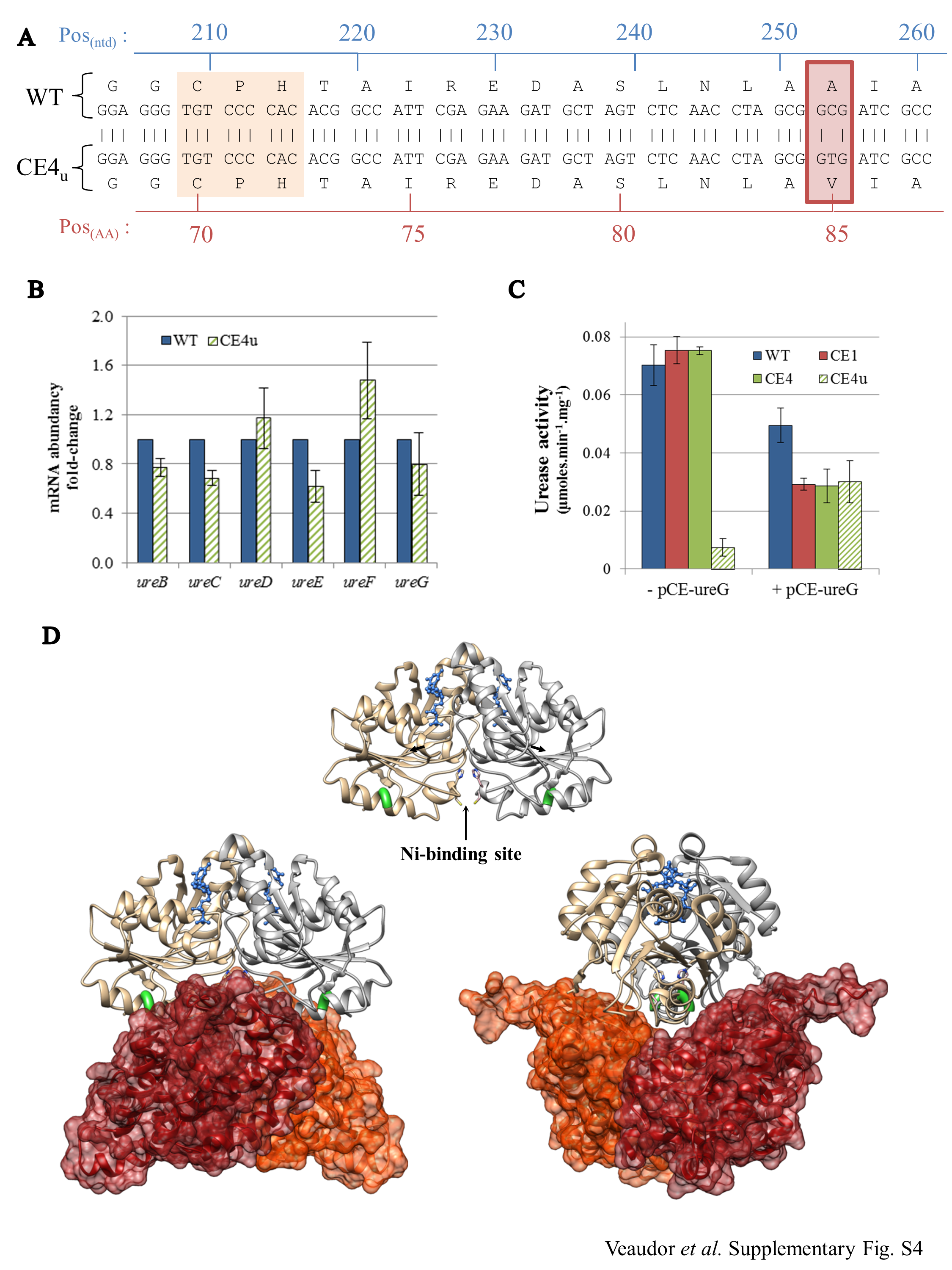

Supplement: S4 Fig — (A) Positions of the nucleotide (blue) and amino-acid (red) sequence of the relevant part of ureG showing the mutation (red rectangle) and conserved nickel binding site (orange rectangle). (B) Histogram representation of transcript abundance (RT-qPCR) of the urease genes in the WT strain (blue rectangles) and CE4u (hatched-green bars) mutant. (C) Urease activity of the WT, CE1, CE4 and CE4u strains (blue, red, green and hatched-green rectangles, respectively) lacking (noted as -) or containing (+) the pCE-ureG replicative plasmid that overexpresses ureG. (D) Structural models of the Synechocystis urease accessory proteins (chaperones) UreG and UreF. Subunits of the UreG dimer (top) are represented in their GDP-bound form and colored in light brown and grey, respectively. The mutated A85 residues are highlighted by thick green coils while GDP molecules are depicted in blue. Nickel-binding residues (Cys and His) are represented as sticks. Black arrows point to the G64 residues (Ni binding site). Front (bottom left) and side (bottom right) views of the UreG dimer interacting with the UreF subunits, colored in orange and red, respectively. (TIFF) [file pone.0198836.s004.tIff]

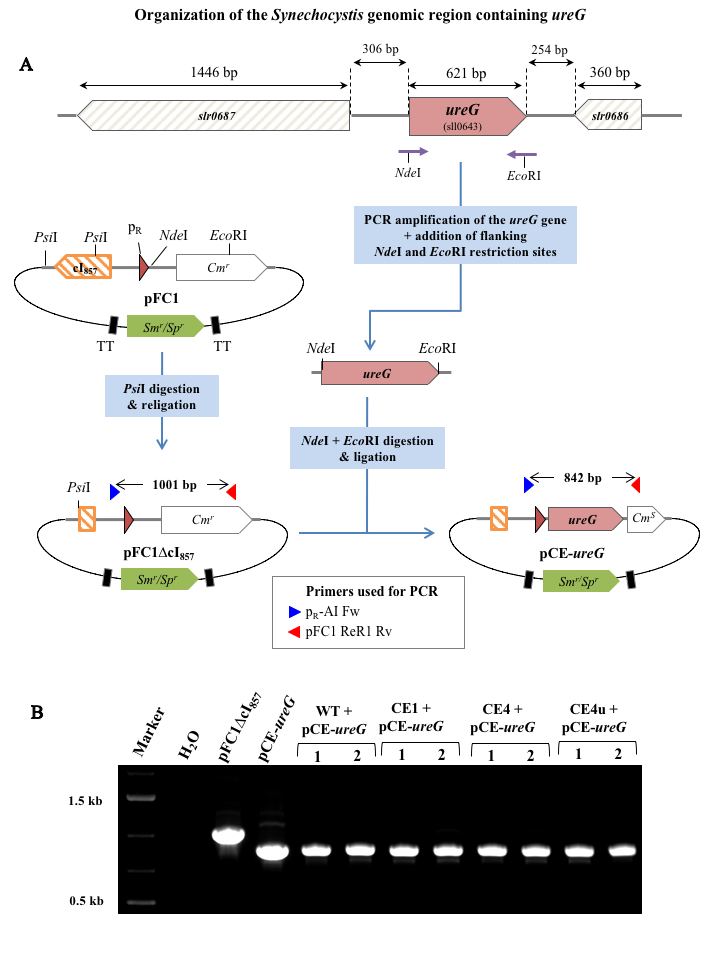

Supplement: S5 Fig — (A) Schematic representation of the Synechocystis chromosome region harboring ureG. The genes are represented by large arrows pointing towards the direction of their transcription. The pFC1 expression vector [13]was digested by PsiI cand religated to inactivate the temperature-dependent λcI857 repressor gene, which normally controls the strong λpR promoter (red triangle), yielding pFC1ΔcI857 (also called pCE, CE for constitutive expression). Meanwhile, ureG was amplified with oligonucleotides primers that introduced a NdeI site (embedding its ATG start codon) and an EcoRI site (behind its stop codon). After NdeI/EcoRI double digestion ureG was cloned between the NdeI and EcoRI sites of pCE, yielding the pCE-ureG plasmid. (B) UV-light image of the agarose gel showing the PCR products typical of the pCE-ureG plasmid replicating in the WT, CE1, CE4 and CE4u strains. Marker (M) = GeneRuler™ 1Kb plus DNA Ladder (Fermentas). The lane noted H2O served as a negative control (no DNA template) while those noted pFC1ΔcI857 and pCE-ureG served as a positive control of the presence of the corresponding plasmid in the studied Synechocystis strains (two clones analyzed in every case). (TIFF) [file pone.0198836.s005.tiff]
