# Supplementary material for: Overproduction of the cyanobacterial hydrogenase and selection of a mutant thriving on urea, as a possible step towards the future production of hydrogen coupled with water treatment
Source: PLoS One. 2018 Jun 7;13(6):e0198836. doi: 10.1371/journal.pone.0198836 (PMC5991728; doi:10.1371/journal.pone.0198836)
Supplement: S1 Table — CS, Protein Coding Sequence; Δ, deletion; TT, transcriptional terminator. (DOCX) [file pone.0198836.s006.docx]

**Table S1.** Characteristics of the plasmids used in this study

| **Plasmid use and name** | **Relevant feature(s)** | **Reference** |
| --- | --- | --- |
| **Cloning and construction of various DNA cassettes** | | |
| pEX-A | Amp^r^ cloning vector | Eurofins |
| pUC19 | Amp^r^ cloning vector | Invitrogen |
| pKNOCK-Gm | Gm^r^ cloning vector | Addgen |
| pUC4K | Source of the Km^r^ cassette without TT | Pharmacia |
| pFC1 | Sm^r /^Spr^r^ Replicating plasmid for temperature-regulated gene expression in cyanobacteria the strong lambda-phage pR promoter and associated ribosome binding site | [[12](#_ENREF_12)] |
| pFC1ΔcI_857_ | Constitutive expression vector corresponding to pFC1 where the cI_857_ repressor gene was truncated after *Psi*I digestion & religation | This study |
| **Targeted deletion of *ureC* and *hoxW* genes in *Synechocystis*** | | |
| pEX-A *ureC* up-dwn | pEX-A containing the upstream and downstream regions of the *Synechocystis* *ureC* gene (sll1750) spaced by a *Sma*I restriction site. | This study |
| pEX-A *ureC*::Km^r^ | pEX-A *ureC* up-dwn with the Km^r^ marker cloned in the *Sma*I site. This plasmid allows to replace the *ureC* CS (its first 971 bp) by the Km^r^ marker | This study |
| pEX-A *hoxW* up-dwn | pEX-A containing the upstream and downstream regions of the *Synechocystis* *hoxW* gene (slr1876) spaced by a *Eco*RV restriction site. | This study |
| pEX-A *hoxW*::Km^r^ | pEX-A *hoxW* up-dwn with the Km^r^ marker cloned in the *Eco*RV site. This plasmid allows to replace the *hoxW* CS (its first 298 bp) by the Km^r^ marker | This study |
| **Cloning of *hoxW* behind the *Synechocystis hoxEFUYH* operon** | | |
| pUC-*hoxW*-Gm^r^ -TT | pUC19 with the *hoxW*-Gm^r^ -TT DNA cassette flanked by *Synechocystis* DNA regions mediating the integration of the *hoxW*-Gm^r^ -TT DNA cassette behind *hoxH* (in place of the 23 bp region behind its stop). | This study |
| **Constitutive overexpression of genes in *Synechocystis*** | | |
| pCE -*hypABCDEF* | Plasmid for high-level expression of the *hypABCDEF* genes in *Synechocystis* | [[13](#_ENREF_13)] |
| pCE-*hoxW* | pFC1ΔcI_857_ with the *Synechocystis hoxW* gene (slr1876) cloned under the control of the strong lambda-phage pR promoter | This study |
| pCE-*ureG* | pFC1ΔcI_857_ with the *Synechocystis ureG* gene (sll0643) cloned under the control of the strong lambda-phage pR promoter | This study |

CS, Protein Coding Sequence; ∆, deletion; TT, transcriptional terminator
