# Supplementary material for: Overproduction of the cyanobacterial hydrogenase and selection of a mutant thriving on urea, as a possible step towards the future production of hydrogen coupled with water treatment
Source: PLoS One. 2018 Jun 7;13(6):e0198836. doi: 10.1371/journal.pone.0198836 (PMC5991728; doi:10.1371/journal.pone.0198836)
Supplement: S2 Table — The restriction sites are written in bold letters; CS, coding sequence; RBS, ribosome binding site. Fw and Rv in the primers names stand for “forward” and “reverse”, respectively. (DOCX) [file pone.0198836.s007.docx]

**Supplementary Table S2** List of the PCR primers used in this study

| **Name** | **Sequence 5’→ 3’** | **Purpose** |
| --- | --- | --- |
| hoxH up *Bam*HI Fw | ATTAAT**GGATCC**GAAGCGGTGGGAGTGAG | Amplification of the *hoxH* upstream region flanked by *Bam*HI and *Xba*I restriction sites |
| hoxH up *Xba*I Rv | GGTAATTTAGGGG**TCTAGA**TTGTTTAATCCCGCTGGATGGACT |  |
| hoxH down *Eco*RV Fw | AGCCAAGG**GATATC**ACAAAAAACATTCAGACGGTC | Amplification of the *hoxH* downstream region flanked by *Eco*RV and *Sal*I sites |
| hoxH down *Sal*I Rv | TTTCCC**GTCGAC**TTTGTCCGCATTGAATCAGTTC |  |
| hoxW SD *Xba*I Fw | **TCTAGA**CCCCTAAATTACCCCAGCTGAGGAGGGAAAATTGAATGCC | Amplification of the *hoxW* CS with a RBS flanked by *Eco*RV and *Xba*I sites |
| hoxW *Eco*RV Rv | TTTGT**GATATC**CCTTGGCTTTATCTCTCCCC |  |
| Gm Fw | TGACATAAGCCTGTTCGG | Amplification of the Gm^r^ cassette |
| Gm TT Rv | CTCGAATTGACGCGTCGGCC |  |
| hox up *Bam*HI Fw | GGCATTGATAAT**GGATCC**CTCGTAGGTCTAA | Assay of chromosome segregation in CE1 mutant |
| hoxE Rv | GCCAATACCGCTTCGTCATTCT |  |
| hoxH up *Bam*HI Fw | ATTAAT**GGATCC**GAAGCGGTGGGAGTGAG | Assay of chromosome segregation in CE4 mutants |
| hox dwn Rv bis | ACCGGACGTCAAGTCTCGTTGACAAAA |  |
| Km *Hinc*II Fw | ACCTGCAGGGG**GTCGAC**GGAAAGCCAC | Amplification of the Km^r^ gene of pUC4K with a *Hinc*II site |
| Km *Hinc*II Rv | GGCGCTGAG**GTCGAC**CTCGTGAAGAAG |  |
| pEX-A Fw | GGAGCAGACAAGCCCGTCAGG | Verification of the Km^r^ cassette in plasmids pEX-A *ureC*::Km^r^ and pEX-A *hoxW*::Km^r^. |
| KmR9 Rv | CGCGGCCTCGAGCAAGACGTTTCCCGTTGAATATGGCTC |  |
| KmA9 Fw | GCTCTCATCAACCGTGGCTCCCTCACTTTCTGGCTGGATGATGGGGCG | Verification of the Km^r^ cassette in plasmids pEX-A *ureC*::Km^r^ and pEX-A *hoxW*::Km^r^. |
| pEX-A Rv | CAGGCTTTACACTTTATGCTTCCGGC |  |
| ureC a Fw | TCGCTGTCTAACTACCACCTTGA | Assay of chromosome segregation in Δ*ureC*::Km^r^ clones |
| ureC b Rv | CGTTTGCCAAGTACGACAGATAA |  |
| hoxW a Fw | GATAGGTTTTGCGGACTGTGCTA | Assay of chromosome segregation in Δ*hoxW*::Km^r^ clones |
| hoxW b Rv | ATCTGGGCTAAGGCTTCGGCTT |  |
| hoxW NdeI Fw | CCAGCTGGTTAACGAAAAT**CATATG**CCAGGCCAAT | Amplification of *hoxW* with a *Nde*I restriction site before its ATG start codon |
| hoxW EcoRI Rv | AGCCTAACTTTTCAATTTT**GAATTC**CCCTTGGCTT | Amplification of *hoxW* with a *Eco*RI site downstream its stop codon |
| ureG NdeI Fw | CCTGTTAGTACCATTTAGT**CATATG**GCTCAAACTC | Amplification of *ureG* with a *Nde*I site before its ATG start codon |
| ureG EcoRI Rv | TAAAAAGGCTCTTAAGCTT**GAATTC**ATTAACTTGC | Amplification of *ureG* with a *Eco*RI site downstream its stop codon |
| pFC1 p_R_-AI Fw | GGCGACGTGCGTCCTCAAGC | Sequencing of the genes (coding sequences) cloned in pFC1-derived plasmids |
| pFC1 ReR1 Rv | GTGTAACAAGGGTGAACAC |  |
| ureA seq Fw | AAAGCATCGTCGCCAAAGCAGTGAT | Verification of the *ureA* gene and 200 bp flanking regions |
| ureA seq Rv | GGCTAGGCAGTCTCGATAACAGTTCGT |  |
| ureB seq Fw | AGTGTGGCTTTGGGGAGTATCTTCGT | Amplification and sequence of the *ureB* gene and 200 bp flanking regions |
| ureB seq Rv | ACCAAATCATTTCCAAAAGCCAACTGC |  |
| ureC seq Fw | GCTGAGATTCAAAGTTTTCGCACCCAAC | Amplification and sequence of the *ureC* gene and 200 bp flanking regions |
| ureC seq Rv | TTGTAATCCTTGACGCAACCAATCGC |  |
| ureD seq Fw | TTGGTCACCTAGAACGATTGGATGC | Amplification and sequence of the *ureD* gene and 200 bp flanking regions |
| ureD seq Rv | AACCAGTTAGATAAAGTTCGGCAAAGCT |  |
| ureE seq Fw | AATCGGGACAGTATCCGCAAGTTAC | Amplification and sequence of the *ureE* gene and 200 bp flanking regions |
| ureE seq Rv | CTGAGGTTGGGAGGATTGAGGTTTGATT |  |
| ureF seq Fw | CTCTGTTGTTGGAGGTATTCACCGATCT | Amplification and sequence of the *ureF* gene and 200 bp flanking regions |
| ureF seq Rv | CTGTAACAGTAGCAGTCATAGGATGAGGT |  |
| ureG seq Fw | ATCTGATAGTGAGGCTTAGGGAGTTCA | Amplification and sequence of the *ureG* gene and 200 bp flanking regions |
| ureG seq Rv | TTATCAGCAGCTTGTTGACTGTCGGT |  |
| rnpB qPCR Fw | GTGAGGACAGTGCCACAGAA | qPCR assay of *rnpB* expression |
| rnpB qPCR Rv | GGCAGGAAAAAGACCAACCT |  |
| rrnA qPCR Fw | CACACTGGGACTGAGACAC | qPCR assay of *rrnA* expression |
| rrnA qPCR Rv | CTGCTGGCACGGAGTTAG |  |
| hoxW qPCR Fw | TATTTCCACCCATCAACTCA | qPCR assay of *hoxW* expression |
| hoxW qPCR Rv | ATTTTAGCCAGGGTCAAGAG |  |
| ureA qPCR Fw | CAAACTCAACTATCCCGAAGCC | qPCR assay of *ureA* expression |
| ureA qPCR Rv | GTCACCAGTTTAGTGCCATCG |  |
| ureB qPCR Fw | TCAAGTGGGTTCCCATTACCAT | qPCR assay of *ureB* expression |
| ureB qPCR Rv | CACCAAGCCATTGAAGCCATAG |  |
| ureC qPCR Fw | GTCAAAGCTGATGTGGGCATTA | qPCR assay of *ureC* expression |
| ureC qPCR Rv | TAAGGCCGTTTCAATCTGTTGG |  |
| ureD qPCR Fw | CGGCGGGAAAGATTTATCGTAG | qPCR assay of *ureD* expression |
| ureD qPCR Rv | AAACGGACAATTTCCCAACCTT |  |

| ureE qPCR Fw | GATAAACCTGGCTTTCCGTCTC | qPCR assay of *ureE* expression |
| --- | --- | --- |
| ureE qPCR Rv | CACATGGCGATTACCCAAATGA |  |
| ureF qPCR Fw | GACACAGCCATTATGGTTCGAG | qPCR assay of *ureF* expression |
| ureF qPCR Rv | TGCACAGTAGGCTCTAAATCCA |  |
| ureG qPCR Fw | GGGAGATAATTTAGCAGCCACC | qPCR assay of *ureG* expression |
| ureG qPCR Rv | TTTGCATCCCGATCCATGATTC |  |

The restriction sites are written in bold letters; CS, coding sequence; RBS, ribosome binding site. Fw and Rv in the primers names stand for “forward” and “reverse”, respectively
